# Neurodegeneration and the ordered assembly of α-synuclein

**DOI:** 10.1007/s00441-017-2706-9

**Published:** 2017-11-08

**Authors:** Maria Grazia Spillantini, Michel Goedert

**Affiliations:** 1 Department of Clinical Neurosciences, Clifford Allbutt Building, University of Cambridge, Cambridge, UK; 2 MRC Laboratory of Molecular Biology, Cambridge, UK

**Keywords:** Alpha-synuclein, Multiple system atrophy, Dementia with Lewy bodies, Parkinson’s disease, Ordered assembly

## Abstract

In 2017, it was 200 years since James Parkinson published ‘An Essay on the Shaking Palsy’ and 20 years since α-synuclein aggregation came to the fore. In 1998, multiple system atrophy joined Parkinson’s disease and dementia with Lewy bodies as the third major synucleinopathy. Here, we describe the work that led to the identification of α-synuclein in Lewy bodies, Lewy neurites and Papp–Lantos bodies. We also review some of the findings reported since 1997.

## Introduction

In 1817, James Parkinson (1755–1824) of Hoxton Square, East London, described the ‘Shaking Palsy’ (Parkinson 1817), a disease that was subsequently named after him (Sanders 1865; Charcot 1875). At the time, the involvement of the substantia nigra and the presence there of Lewy pathology were not known.

Paul Blocq (1860–1896) and Georges Marinesco (1863–1938) of the Salpêtrière Hospital in Paris reported a patient with left-sided parkinsonian tremor who, at autopsy, had an enucleated tuberculoma the size of a hazelnut in the right substantia nigra (Blocq and Marinesco 1893). They also alluded to a case from Jean-Martin Charcot (1825–1893) with hemiparkinsonism caused by a tumour that compressed the cerebral peduncle. This led Edouard Brissaud (1852–1909), Charcot’s successor at the Salpêtrière, to propose, in 1894, that a lesion of the substantia nigra was the anatomical substrate of Parkinson’s disease (PD) (Brissaud and Meige 1895).

In 1919, at the Salpêtrière, Constantin Trétiakoff (1892–1956) reported pathological inclusions that he named ‘corps de Lewy’ in the substantia nigra in PD (Trétiakoff 1919) [similar inclusions had been identified in other brain areas of PD by Fritz Jakob Heinrich Lewy (1885–1950) (Lewy 1912; Goedert et al. 2013)]. Trétiakoff also showed degeneration of the substantia nigra and postulated a link between nerve cell loss, rigidity and tremor. Rolf Hassler (1914–1984) confirmed Trétiakoff’s findings and showed that the ventrolateral tier was the most severely affected part of the substantia nigra (Hassler 1938). He did most of this work at the Kaiser Wilhelm Institute for Brain Research in Berlin, which was directed by Oskar Vogt. Following their dismissal in 1936, Vogt and his wife Cécile built up a new Institute in Neustadt in the Black Forest, where Hassler worked for a number of years. Science is often a young person’s game. Lewy, Marinesco, Trétiakoff and Hassler were 30 years old or less when they made these discoveries.

In 1997, the ordered assembly of α-synuclein came to the fore (Polymeropoulos et al. 1997; Spillantini et al. 1997). Polymeropoulos et al. described a causative mutation (A53T) in *SNCA*, the α-synuclein gene, in the Contursi kindred and three smaller Greek families with PD, whereas Spillantini et al. reported the presence of α-synuclein in Lewy bodies and Lewy neurites of idiopathic PD and dementia with Lewy bodies (DLB). These findings linked the genetic cause of a rare form of PD with the inclusions in all cases of the disease. They were conceptually similar to those previously obtained in Alzheimer’s disease (AD) (Glenner and Wong 1984; Goate et al. 1991) and some human tauopathies (Pollock et al. 1986; Poorkaj et al. 1998; Hutton et al. 1998; Spillantini et al. 1998a) and helped to underscore the view expressed by William Harvey (1578–1657) and reiterated by Archibald Garrod (1857–1936), that the study of rare forms of disease can inform the more common cases (Garrod 1928). In his letter of April 24, 1657, to John Vlackveld, Harvey wrote: “Nature is nowhere accustomed more openly to display her secret mysteries than in cases where she shows traces of her workings apart from the beaten path; nor is there any better way to advance the proper practice of medicine than to give our minds to the discovery of the usual law of nature, by the careful investigation of cases of rarer forms of disease.” (Harvey and Willis 1847).

## α-Synuclein and Lewy pathology

**Fig. 1:**
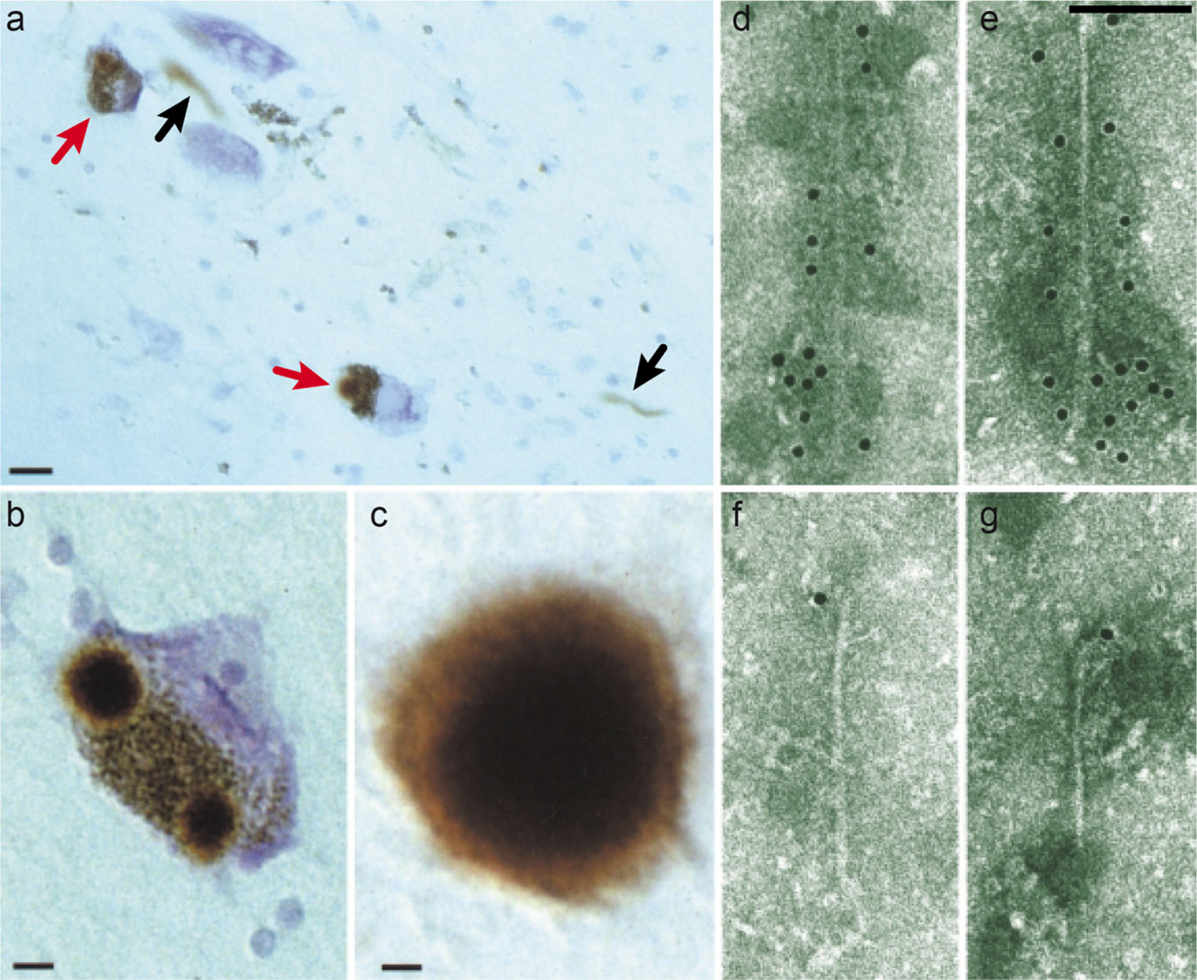
The α-synuclein pathology of Parkinson’s disease. Lewy pathology in the substantia nigra and several other brain regions defines Parkinson’s disease at the neuropathological level. This is shown by light microscopy, labelled by α-synuclein antibodies (**a**–**c**). Using immunoelectron microscopy, filaments extracted from the Lewy pathology were decorated by α-synuclein antibodies (**d**–**g**). **a** Two pigmented nerve cells, each containing an α-synuclein-positive Lewy body (*red arrows*); Lewy neurites (*black arrows*) are also immunopositive. *Scale bar* 20 μm. **b** Pigmented nerve cell with two α-synuclein-positive Lewy bodies. *Scale bar* 8 μm. **c** α-Synuclein-positive extracellular Lewy body. *Scale bar* 4 μm. **d**–**g** Isolated filaments from the substantia nigra of patients with Parkinson’s disease are decorated by an antibody directed against the carboxy-terminal (**d**, **e**) or the amino-terminal (**f**, **g**) region of α-synuclein. The gold particles conjugated to the second antibody appear as *black dots*. Note the uniform decoration (**d**, **e**) and the labelling of only one filament end (**f**, **g**). *Scale bar* 100 nm. From Goedert (2001)

Our findings on α-synuclein (Jakes et al. 1994) grew out of work on tau, which we found to be an integral component of the paired helical and straight filaments of AD in 1988 (Goedert et al. 1988; Wischik et al. 1988a, b). In August 1997, together with Ross Jakes, Marie-Luise Schmidt, Virginia Lee and John Trojanowski, we showed that the Lewy pathology from the substantia nigra of six patients with idiopathic PD and four patients with DLB was strongly immunoreactive for α-synuclein (Fig. 1a–c) (Spillantini et al. 1997). The same was true of the Lewy pathology from the cingulate cortex of DLB. Antibodies specific for the amino- and carboxy-termini of α-synuclein stained the inclusions, consistent with the presence of the whole molecule. An antibody specific for β-synuclein failed to label the inclusions of PD and DLB.

In May 1998, we reported that Lewy neurites were more abundant in PD and DLB than hitherto believed (Spillantini et al. 1998b). The staining of intraneuritic Lewy bodies helped to reinforce the view that the Lewy pathology is not benign. Prior to this work, ubiquitin staining had been the most sensitive means of detecting Lewy pathology but it lacked in specificity, because inclusions made of other proteins can also be ubiquitinated. We showed that staining for α-synuclein was more extensive than staining for ubiquitin, indicating that the assembly of α-synuclein precedes ubiquitination. Similar findings were subsequently reported by others (Hasegawa et al. 2002; Sampathu et al. 2003).

We confirmed that β-synuclein did not accumulate in the Lewy pathology and showed that γ-synuclein was not present either. Of the three mammalian synucleins, only α-synuclein is found in the Lewy pathology. We then studied sarkosyl-insoluble filaments extracted from the cingulate cortex of patients with DLB by immunoelectron microscopy. An antibody specific for the carboxy-terminal region of α-synuclein labelled filaments with a diameter of 5–10 nm and a length of 200–600 nm. An antibody specific for the amino-terminal region only labelled one end of each filament, suggesting that α-synuclein filaments are polar structures. We subsequently reported similar findings on filaments from the substantia nigra of PD patients (Fig. 1d–g) (Crowther et al. 2000).

## α-Synuclein and Papp-Lantos bodies

Multiple system atrophy (MSA) is a neurodegenerative disease characterised by a combination of autonomic, cerebellar, parkinsonian, pyramidal and cognitive symptoms (Goedert 2015). It is divided into parkinsonian (MSA-P) and cerebellar (MSA-C) variants. A rarer cortical variant (MSA-FTLD) has also been described. In most countries, MSA-P is the most common form. MSA comprises what used to be called olivopontocerebellar atrophy, striatonigral degeneration and Shy–Drager syndrome.

**Fig. 2:**
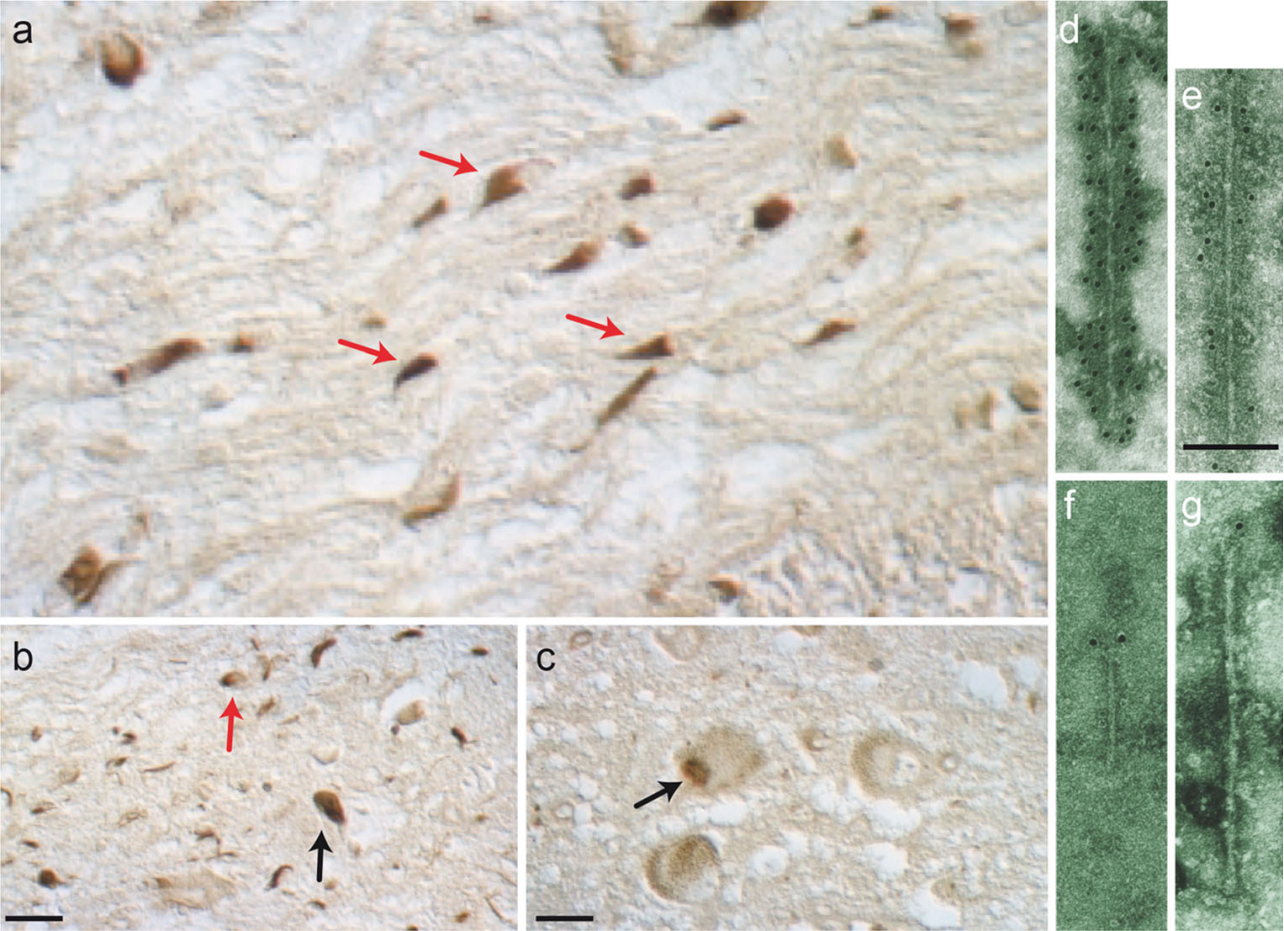
The α-synuclein pathology of multiple system atrophy. Glial cytoplasmic inclusions in several brain regions define multiple system atrophy. Similar inclusions are also present in the nuclei of some glial cells, as well as in the cytoplasm and nuclei of some nerve cells and in nerve cell processes. Inclusions labelled by α-synuclein antibodies are shown by light microscopy (**a**–**c**). Using immunoelectron microscopy, filaments extracted from the inclusions were decorated by α-synuclein antibodies (**d**–**g**). **a** α-Synuclein-immunoreactive cytoplasmic oligodendrocyte inclusions (*red arrows*) in pontine fibre tracts. **b** α-Synuclein-immunoreactive nuclear oligodendrocyte inclusion (*red arrow*) and cytoplasmic nerve cell inclusion (*black arrow*) in grey matter of frontal cortex. **c** α-Synuclein-immunoreactive nuclear nerve cell inclusion (*black arrow*) in grey matter of pons. *Scale bars* (**a**) 5 μm, (**b**) 50 μm, (**c**) 30 μm. **d**–**g** Isolated filaments from the frontal cortex and cerebellum of patients with multiple system atrophy are decorated by antibodies specific for the carboxy-terminal (**d**, **e**) and amino-terminal (**f**, **g**) regions of α-synuclein. The gold particles conjugated to the second antibody appear as *black dots*. Note the uniform decoration in (**d**, **e**) and the labelling of only one filament end in (**f**, **g**). A twisted filament is shown in (**d**), whereas (**e**) shows a straight filament. *Scale bar* 100 nm. Adapted from Goedert (2001)

Inclusions in the cytoplasm of oligodendrocytes (Papp–Lantos bodies) are the major histological hallmark of MSA (Papp et al. 1989). Less often, nuclear inclusions are present, as are neuronal cytoplasmic and nuclear inclusions. Together with Nigel Cairns and Peter Lantos at the Institute of Psychiatry of King’s College London, we showed that glial and neuronal inclusions of MSA contain α-synuclein (Fig. 2a–c) (Spillantini et al. 1998c). The inclusions were stained by antibodies recognising the amino- and carboxy-termini of α-synuclein. By double-labelling, staining for α-synuclein was more extensive than staining for ubiquitin, indicating that the aggregation of α-synuclein preceded ubiquitination. Antibodies against β- and γ-synuclein failed to stain the inclusions. Similar results were reported by others at about the same time (Wakabayashi et al. 1998; Tu et al. 1998).

**Fig. 3:**
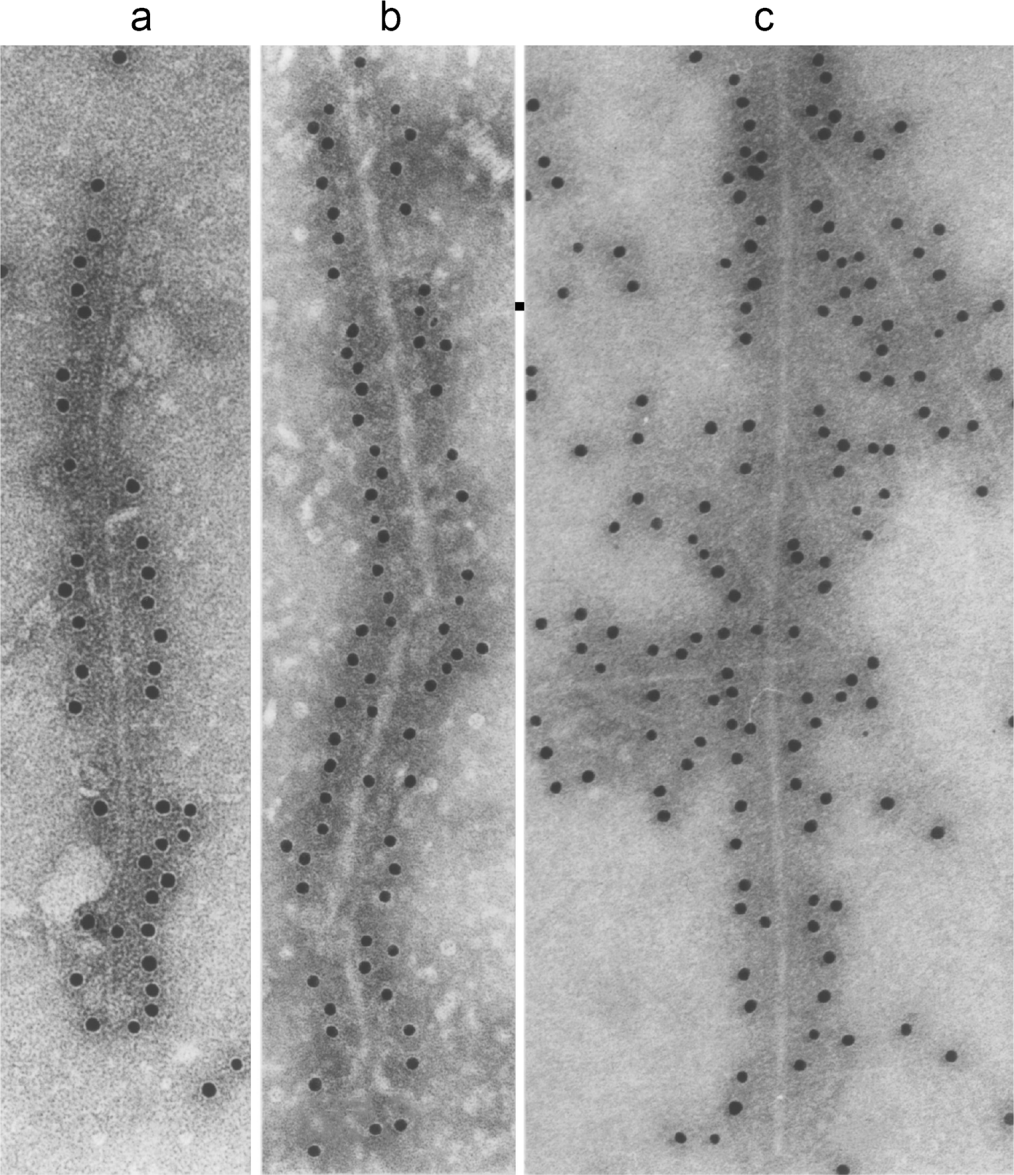
Filaments extracted from the brains of patients with dementia with Lewy bodies (**a**), multiple system atrophy (**b**) or assembled from bacterially expressed human α-synuclein (**c**) were decorated by an anti-α-synuclein antibody. The gold particles conjugated to the second antibody appear as *black dots*. From Goedert and Spillantini (2012)

Filaments from MSA brains had a diameter of 5–18 nm and were strongly labelled by an antibody specific for the carboxy-terminus of α-synuclein (Fig. 2d, e) (Spillantini et al. 1998c). An amino-terminal antibody only labelled one filament end, as was the case in PD and DLB (Fig. 2f, g). This work revealed a molecular link between MSA and Lewy pathology disorders. However, unlike PD and DLB, where α-synuclein filaments are mostly present in the cytoplasm of nerve cells in the form of Lewy bodies and Lewy neurites, in MSA, they are found in the cytoplasm and nuclei of both nerve cells and glial cells. Since 1998, PD, DLB and MSA have frequently been called ‘synucleinopathies’ (Goedert and Spillantini 1998). Filaments assembled from bacterially expressed human α-synuclein are structurally and antigenically similar to those extracted from DLB and MSA brains (Fig. 3) (Crowther et al. 1998; Conway et al. 1998). However, higher-resolution techniques, such as cryogenic electron microscopy, may reveal structural differences between these filaments in the future.

## Twenty years of synucleinopathies

Lewy pathology is also the defining feature of several rarer diseases, including pure autonomic failure, in which α-synuclein aggregates in the peripheral sympathetic nervous system are the major neuropathological hallmark (Arai et al. 2000). In PD, abundant Lewy pathology is present in the enteric, peripheral and central nervous systems. Some patients presenting clinically with pure autonomic failure go on to develop PD or DLB (Kaufmann et al. 2004). In incidental Lewy body disease, which may be a preclinical form of PD, Lewy pathology is present in the absence of clinical motor symptoms (Iwanaga et al. 1999; Del Tredici et al. 2002; Dickson et al. 2008; Beach et al. 2008). Similarly, cases with oligodendroglial α-synuclein inclusions in the absence of clinical symptoms, akin to preclinical MSA, have been described (Parkkinen et al. 2007; Fujishiro et al. 2008).

The clinical Parkinson’s syndrome is defined as bradykinesia that worsens over time, in conjunction with at least one of three additional features: rigidity, resting tremor or gait disturbance (Jenner et al. 2013; Postuma et al. 2015). At the time of diagnosis, around 30% of dopaminergic neurons in the substantia nigra and 50–60% of their axon terminals have been lost (Cheng et al. 2010), consistent with a centripetal mechanism of aggregate formation and neurodegeneration.

### Physiological function of α-synuclein

The physiological function of α-synuclein is incompletely understood. It binds to acidic phospholipids through its amino-terminal repeats (Davidson et al. 1998; Jensen et al. 1998), when it multimerizes and becomes α-helical (Chandra et al. 2003; Ulmer et al. 2005; Jao et al. 2008). About 3500 α-synuclein molecules co-exist with 300 synaptic vesicles in individual synaptic boutons from rat brain (Wilhelm et al. 2014). The presence of α-synuclein in nerve terminals has suggested a role in neurotransmitter release. It has been reported that it promotes dilation of the exocytic fusion pore (Logan et al. 2017). Mitochondria fragment upon α-synuclein expression (Kamp et al. 2010; Nakamura et al. 2011), despite the fact that in nerve cells α-synuclein is concentrated in nerve terminals, whereas most mitochondria localise to nerve cell bodies and dendrites (Bendor et al. 2013).

Loss of *SNCA* does not lead to a neurodegenerative phenotype (Abeliovich et al. 2000). The existence of three synucleins raised the possibility that redundancy might account for the relatively mild *SNCA* knockout phenotype. Mice lacking α-, β- and γ-synucleins were subsequently produced (Greten-Harrison et al. 2010; Anwar et al. 2011). They showed an increase in striatal dopamine release beyond that of single knockouts, probably because synaptic vesicles fused more with presynaptic membranes. However, the overall phenotype was relatively mild. No synuclein homologues are found in *C. elegans* or *D. melanogaster*.

### α-Synuclein inclusions

The core of an α-synuclein filament, which is the sequence required for a filament from human brain to look like a filament by electron microscopy, extends over approximately 70 amino acids (residues 30–100) (Miake et al. 2002; Der-Sarkissian et al. 2003). The crystal structure of residues 68–78 of human α-synuclein showed paired β-sheets with parallel β-strands in each sheet and anti-parallel β-strands between the sheets. The zipper structure that marked the region between paired sheets was longer than in other peptide structures, and each pair of β-sheets contained two water molecules (Rodriguez et al. 2015). Upon assembly, full-length α-synuclein adopts structures rich in β-sheets (Serpell et al. 2000). Recombinant α-synuclein that had been aggregated using a seed from PD brain was studied by solid-state nuclear magnetic resonance, scanning transmission electron microscopy and X-ray diffraction (Tuttle et al. 2016). The core of the filament (residues 44–97) consisted of parallel in-register β-sheets with the topology of a Greek key.

Assembly of α-synuclein is nucleation-dependent. Deletion of residues 71–82 abolished the ability to assemble into filaments (Giasson et al. 2001); these residues are located in the innermost β-sheet of the core (Tuttle et al. 2016). Deletion of residues 66–74 also prevented assembly (Du et al. 2003), whereas the absence of the carboxy-terminal region promoted assembly (Crowther et al. 1998).

### Genetics of SNCA

**Fig. 4:**
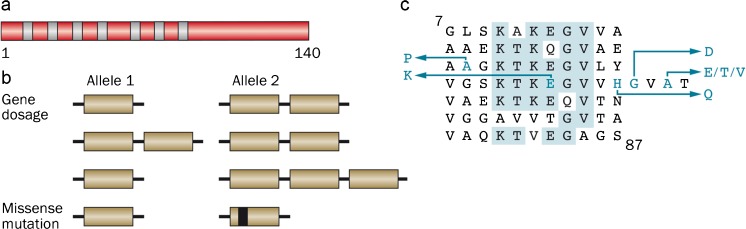
Human α-synuclein and its disease-causing mutations. **a** Diagram of the 140 amino acid human α-synuclein. The seven amino-terminal repeats are shown as *blue bars*. **b** A dominantly inherited increase in gene dosage (duplication or triplication) of the chromosomal region containing *SNCA* gives rise to Parkinson’s disease and dementia with Lewy bodies. Homozygous duplications have also been described. In addition, missense mutations in *SNCA* cause dominantly inherited forms of Parkinson’s disease and dementia with Lewy bodies. **c** The repeats (residues 7–87) of human α-synuclein are shown, with disease-causing mutations (A30P, E46K, H50Q, G51D, A53E, A53T and A53V) given as *blue letters*. Amino acids that are identical in at least five of the seven repeats are shaded in *blue*

Seven dominantly inherited missense mutations in *SNCA* have been described as the cause of familial PD (Fig. 4). Besides A53T, they include A30P (Krüger et al. 1998), E46K (Zarranz et al. 2003), H50Q (Appel-Cresswell et al. 2013; Proukakis et al. 2013), G51D (Kiely et al. 2013; Lesage et al. 2013), A53E (Pasanen et al. 2014; Martikainen et al. 2015) and A53V (Yoshino et al. 2017). The age of disease onset can be variable, even within families but mutations G51D, A53E and A53T give rise to the earliest onset. Experimentally, mutations E46K, H50Q and A53T increase α-synuclein inclusion formation (Serpell et al. 2000; Choi et al. 2004; Ghosh et al. 2013), whereas mutations A30P, G51D and A53E (Narhi et al. 1999; Bilal-Fares et al. 2014; Ghosh et al. 2014; Rutherford et al. 2014) reduce aggregation rates. Mutations A30P, G51D and A53E also lead to a reduced ability of mutant α-synuclein to interact with acidic phospholipids (Chandra et al. 2003; Bilal-Fares et al. 2014; Ghosh et al. 2014; Ysselstein et al. 2015). These findings are consistent with work that has suggested an antagonistic relationship between lipid binding of α-synuclein and aggregation into cytotoxic species (Burré et al. 2015; Iljina et al. 2016; Cremades et al. 2012).

Dominantly inherited duplications and triplications of the chromosomal region that contains *SNCA* have also been found to cause PD (Fig. 4) (Singleton et al. 2003; Chartier-Harlin et al. 2004; Ibánez et al. 2004). A homozygous duplication of *SNCA* has been described (Ikeuchi et al. 2008). The sequence of α-synuclein was wild-type, showing that an increase in protein levels rather than a change in its properties is sufficient to cause PD. Heterozygous duplications of *SNCA* gave rise to a form of PD that was similar to the sporadic disorder in terms of age of onset and symptoms but triplication caused a more severe phenotype, with an earlier age of onset and prominent cognitive impairment.

Individuals with the A53T mutation in *SNCA* developed a severe form of PD that was often accompanied by dementia. A clinical picture resembling DLB was characteristic of a family with the E46K mutation, whereas individuals from the family with the A30P mutation developed late-onset PD and had only mild dementia. Neuropathologically, some individuals, in particular those with mutations G51D and A53E, had features of both PD and MSA. This overlap of clinical and neuropathological characteristics supports the view that the aetiologies of PD, DLB and MSA are closely related.

Heterozygous mutations in the gene encoding leucine-rich repeat kinase 2 (*LRRK2*) are the most common cause of dominantly inherited PD (Paisán-Ruiz et al. 2004; Zimprich et al. 2004). LRRK2 is a multidomain protein of 2527 amino acids with two enzymatic activities (guanosine triphosphatase and protein kinase) and multiple protein–protein interaction domains. G2019S, the most common mutation, increases LRRK2’s kinase activity 2- to 3-fold. Disease penetrance is incomplete. Some Rab GTPases are prominent LRRK2 targets (Steger et al. 2016) and their increased phosphorylation may result in disturbed vesicle trafficking. Moreover, mutations in the gene encoding TMEM230, a transmembrane protein of synaptic vesicles, give rise to inherited PD (Deng et al. 2016). The resulting impairment of vesicle trafficking may impair the degradation of α-synuclein, resulting in a net effect not unlike that of gene dosage mutations.

Genome-wide association studies (GWAS) of risk in idiopathic PD showed that *SNCA* makes the largest contribution. The implicated polymorphisms lie outside the coding region and thus probably affect mRNA expression, resulting in increased expression of α-synuclein (Satake et al. 2009; Simón-Sánchez et al. 2009; Nalls et al. 2014). Variability in *LRRK2*, *GAK* (cyclin G-associated kinase) and *MAPT* (microtubule-associated protein tau) has also been implicated. Variants in *SNCA* and *MAPT* have been reported as risk factors for MSA (Scholz et al. 2009; Al-Chalabi et al. 2009; Vilarino-Güell et al. 2011). However, a GWAS of risk in MSA failed to confirm these findings (Sailer et al. 2016). None of the studied variants were statistically significant. The estimated heritability of MSA is lower than that of PD (Federoff et al. 2016).

The most common genetic risk factor for idiopathic PD and DLB, missense mutations in one or both alleles of *GBA1*, the glucocerebrosidase gene, was not discovered using GWAS but through clinical studies (Neudorfer et al. 1996; Aflaki et al. 2017). *GBA1* encodes glucocerebrosidase, which degrades glucosylceramide into glucose and ceramide. Homozygous loss-of-function mutations in *GBA1* cause Gaucher’s disease, a lysosomal storage disorder. Approximately 7% of patients with PD carry mutations in *GBA1*. Conversely, 5–7% of patients with Gaucher’s disease develop PD before the age of 70. The mechanistic links between glucocerebrosidase and α-synuclein are unclear but there appears to be an inverse correlation between the levels of glucocerebrosidase and α-synuclein (Mazzulli et al. 2011). Experimental evidence supports a direct interaction between α-synuclein and glucocerebrosidase (Yap et al. 2011). Mutations in *GBA1* may also predispose to MSA (Mitsui et al. 2015).

### Propagation of α-synuclein aggregates

Evidence for the existence of prion-like mechanisms in diseased human brain has come from the development of scattered Lewy pathology in foetal human midbrain neurons that were therapeutically implanted into the striata of patients with advanced PD (Li et al. 2008; Kordower et al. 2008). Lewy pathology was detected in 2–5% of grafted cells 10 or more years after transplantation, approximately the same percentage as that of neurons with Lewy pathology in the pars compacta of the substantia nigra in PD. After 24 years, 11–12% of grafted dopaminergic neurons exhibited α-synuclein- and ubiquitin-positive inclusions (Li et al. 2016).

Over the past 8 years, experimental studies have shown that the intracerebral injection of α-synuclein assemblies from diseased human brains or recombinant proteins induces nerve cells to form intracellular inclusions at the injection sites, from where they can spread to distant brain regions (Goedert 2015; Shimozawa et al. 2017). Moreover, the peripheral injection of α-synuclein aggregates assembled from recombinant protein caused α-synuclein pathology and neurodegeneration in the central nervous system of transgenic but not wild-type, mice (Breid et al. 2016; Ayers et al. 2017). Using long-term in vivo imaging, aggregated recombinant α-synuclein was shown to seed the ordered assembly of expressed α-synuclein in brain (Osterberg et al. 2015). Inclusion-bearing neurons degenerated, demonstrating that inclusion formation was linked to cellular toxicity. In the substantia nigra from PD patients, the proportion of Lewy body-containing neurons is approximately 4%. The inclusions are probably degraded when the neurons that bear them die. In a model in which neurons are killed by the Lewy pathology, it has been estimated that the mean survival time of an eosinophilic Lewy body is of the order of 6 months (Greffard et al. 2010).

Some morphological differences between disease-associated α-synuclein filaments have been described (Spillantini et al. 1998b). Lewy pathology was positive by Campbell–Switzer silver but not Gallyas–Braak silver (Uchihara et al. 2005a). The same has been shown to be the case of inclusions made of tau isoforms with 3 repeats (Uchihara et al. 2005b). By contrast, the glial cytoplasmic inclusions of MSA were positive by both Campbell–Switzer and Gallyas–Braak silver, like inclusions made of all 6 tau isoforms. Inclusions made of tau isoforms with 4 repeats are only positive with Gallyas–Braak silver. Brain extracts from MSA patients propagated in heterozygous mice transgenic for human A53T α-synuclein, in contrast to brain extracts from PD patients (Woerman et al. 2015; Prusiner et al. 2015). However, unlike in MSA, α-synuclein inclusions were exclusively neuronal.

Despite an increased understanding of the pathogenesis of MSA, the origin of glial α-synuclein aggregates is still unclear. Ordered assembly is concentration-dependent and, until recently, it was believed that mature oligodendrocytes did not express α-synuclein. However, a study based on single-cell capture and quantitative real-time PCR has challenged this view (Asi et al. 2014). It pointed to the possibility that α-synuclein aggregates characteristic of GCIs might be of oligodendroglial origin. Cell-to-cell transfer might also play a role, since oligodendrocytes have been shown to take up α-synuclein assemblies (Kisos et al. 2012; Reyes et al. 2014). It remains to be seen if MSA is a primary gliopathy with neurons involved secondarily, or if it is a primary neuronal problem with glial cells affected secondarily.

Polymorphs of recombinant aggregated α-synuclein in the form of ribbons and fibrils have been described (Bousset et al. 2013). When injected into the rat substantia nigra, the ribbons gave rise to Lewy pathology, whereas the fibrils, which did not seed Lewy pathology, led to the loss of dopaminergic neurons (Peelaerts et al. 2015). It remains to be seen if ribbons and fibrils have their counterparts in human synucleinopathies. In a separate work, some α-synuclein filaments seeded both tau and α-synuclein aggregation, whereas others only seeded α-synuclein aggregation (Guo et al. 2013). These conformers of aggregated α-synuclein exhibited different properties after proteinase K digestion. They were similar to prion strains, in that they showed structural variations, differences in seeding properties and heritability of phenotypic traits.

## Conclusion

The ordered assembly of α-synuclein has proved to be central to PD, DLB and MSA. Understanding disease aetiology and pathogenesis will probably be necessary for the development of safe and effective mechanism-based therapies that are superior to what is currently available. This is a tall order for PD, where L-DOPA has proved to be a good symptomatic therapy for the motor symptoms, at least for some time during the course of the disease. Perhaps future treatments aimed at slowing down or arresting the progression of PD will be complementary to L-DOPA. Although the diagnosis of PD relies on the motor effects of a deficient function of the substantia nigra, there are also non-motor symptoms, such as hyposmia (Ansari and Johnson 1975), REM sleep behaviour disorder (Schenck et al. 1986), depression and constipation, which can precede the motor symptoms by several years (Schapira and Tolosa 2010). The presence of early non-motor features has given rise to the concept of prodromal PD (Berg et al. 2015). The hope is that in the future it will become possible to identify those who are in the preclinical phase of PD, with some α-synuclein inclusions but no symptoms. If so, preventive strategies, when available, could be tried.
